# Genomic profiles of a hepatoblastoma from a patient with Beckwith-Wiedemann syndrome with uniparental disomy on chromosome 11p15 and germline mutation of APC and PALB2

**DOI:** 10.18632/oncotarget.20515

**Published:** 2017-08-24

**Authors:** Shinn Young Kim, Seung-Hyun Jung, Min Sung Kim, Mi-Ryung Han, Hyeon-Chun Park, Eun Sun Jung, Sung Hak Lee, Sug Hyung Lee, Yeun-Jun Chung

**Affiliations:** ^1^ Department of Precision Medicine Research Center, College of Medicine, The Catholic University of Korea, Seoul, South Korea; ^2^ Department of Cancer Evolution Research Center, College of Medicine, The Catholic University of Korea, Seoul, South Korea; ^3^ Department of Pathology, College of Medicine, The Catholic University of Korea, Seoul, South Korea; ^4^ Department of Integrated Research Center for Genome Polymorphism, College of Medicine, The Catholic University of Korea, Seoul, South Korea; ^5^ Department of Microbiology, College of Medicine, The Catholic University of Korea, Seoul, South Korea; ^6^ Department of Surgery, College of Medicine, The Catholic University of Korea, Seoul, South Korea; ^7^ Department of Hospital Pathology, College of Medicine, The Catholic University of Korea, Seoul, South Korea

**Keywords:** hepatoblastoma, uniparental disomy, mutation, copy number alteration, BWS

## Abstract

Beckwith-Wiedemann syndrome (BWS) is a congenital overgrowth disorder mainly associated with altered genomic imprinting at chromosome 11p15.5. Children with BWS, especially uniparental disomy (UPD) at 11p15.5, are at increased risk of embryonal tumors including hepatoblastoma. Although genetic alterations of sporadic hepatoblastomas have been identified, integrated germline and somatic alterations of BWS-related hepatoblastoma have not been reported. For this, we performed whole-exome sequencing and genome-wide loss of heterozygosity/copy number analyses using a single nucleotide polymorphism (SNP) array for a hepatoblastoma in a BWS infant with paternal UPD at chromosome 11p15.5. We found germline 11p15.5 UPD as well as germline mutations of *APC* and *PALB2* in the patient. At the somatic level, we found a *CTNNB1* hotspot mutation and chromosome 1q gain in the tumor. The development of hepatoblastoma in this case might be explained by predisposition of the germline events (11p15.5 UPD, mutations of *APC* and *PALB2*) and later by somatic events with *CTNNB1* somatic mutation and 1q gain. To our knowledge, this is the first report of germline and somatic genomic alteration profiles in hepatoblastoma arising from BWS. Clinically, our results provide a rationale for performing a more strict and intense protocol for hepatoblastoma surveillance in a high-risk BWS infant, such as the UPD-carrying case, for early detection and treatment.

## INTRODUCTION

Beckwith-Wiedemann syndrome (BWS, OMIM 130650), either sporadic (>85%) or familial (<15%), is caused by mutation or deletion of imprinted genes *CDKN1C*, *H19* and *LIT1* as well as by hypermethylation in the H19/IGF2-imprinting control region within the chromosome 11p15.5 region [1]. Some other sporadic BWSs harbor paternal uniparental disomy (UPD) that results in the replacement of the maternal 11p15.5 with an extra paternal copy. About 20% of BWS patients have paternal UPD [2]. UPD occurs in BWS as a postfertilization mitotic recombination event that results in somatic mosaicism [3]. Patients with BWS are characterized by phenotypic presentations of overgrowth including macrosomia, macroglossia, ear defects and anterior abdominal wall defects as well as severe hypoglycemia [2].

The incidence of tumors in BWS patients is estimated to be 7.5% (range 4-21%), which is far higher (relative risk of 676) than that in other children [4]. Such tumors include Wilms’ tumor (43%), hepatoblastoma (20%) and adrenocortical carcinoma (7%), and usually occur before 4 years of age (∼90%) [5]. BWS is caused by 11p15.5 alterations that may lead to tissue overgrowth for the phenotypic presentations and may also provide genetic backgrounds for tumor development. However, because most BWS patients with the 11p15.5 alterations do not develop tumors, it is possible that there might be other genetic factors that predispose to tumor development. Hepatoblastoma accounts for approximately 1% of childhood tumors but is the most common primary tumor in childhood liver [6]. It sometimes develops in patients with familial diseases including familial adenomatous polyposis (FAP) and BWS, but usually occurs as sporadic cases [7]. In sporadic hepatoblastomas even without FAP manifestations, *de novo* germline *APC* mutations are found [8].

Somatic mutations are crucial in the development of both hereditary and sporadic tumors. Recent whole-exome sequencing (WES)-based mutation studies identified high frequencies of somatic mutations of *APC*, *β-catenin* (*CTNNB1*: around 80%) and *NFE2L2* (10%), as well as germline *APC* mutations (60%) in hepatoblastomas [9–11]. To our knowledge, only one case of hepatoblastoma in a BWS patient (11p15.5 alteration type was not available) has been studied by WES [9]. This analysis revealed a somatic *CTNNB1* mutation, but no germline *APC* mutation.

To further extend the knowledge on BWS-associated hepatoblastoma development, we performed WES of a hepatoblastoma in a BWS infant with paternal UPD on chromosome 11p15.5 and germline *APC* mutation in this study.

## RESULTS

### Clinical feature of the patient

An infant boy was born by caesarian section at gestational age of 38 weeks due to his intrauterine overgrowth. Apgar score was 6 at 1 minute and 8 at 5 minutes. He had macroglossia and macrosomia. His weight was 4.825 kg (>90 percentile), height was 53 cm (90 percentile), and head conference was 34 cm (50 percentile). His initial blood sugar level was 17 mg/dl (neonatal hypoglycemia), which was recovered with glucose injection by the third day of birth. Presence of three of the five common features associated with BWS (macroglossia, macrosomia, midline abdominal wall defects, ear creases/ear pits, and neonatal hypoglycemia) prompted the diagnosis as BWS. His parents as well as the second and third degree relatives did not have any evidence to suspect BWS. They did not have histories of FAP nor hepatoblastoma.

**Figure 1:**
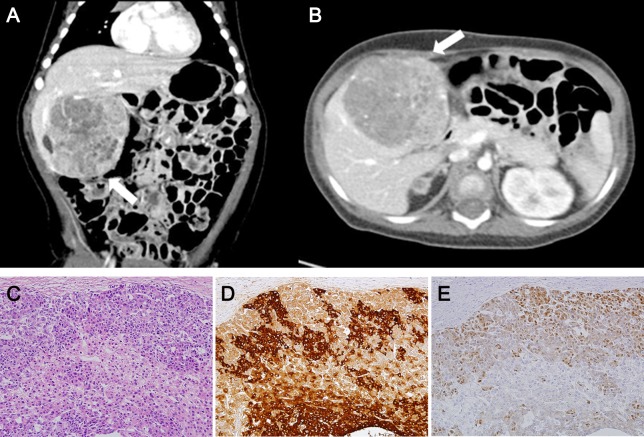
Abdominal computer tomography and histopathology of the case Abdominal computer tomography scan at 8 months (coronary **(A)** and axial **(B)** view) showing a huge and well-defined heterogeneous mass suspicious of hepatoblastoma classified as PRETEXT stage II (arrow). Hematoxylin and Eosin staining **(C)**, Immunohistochemistry for alpha fetoprotein **(D)** and β-catenin **(E)** show compatible features of hepatoblastoma.

The baby was discharged at 1 month of age with a 3-month tumor screening schedule by abdominal ultrasonography and serum alpha-fetoprotein (AFP) as described elsewhere [12]. On his first visit to the outside clinic (+1 week after discharge), the AFP level was 6,428 ng/ml, which was decreasing compared to the initial AFP level of 124,704 ng/ml (Supplementary Figure 1). On the second visit (+9 weeks), it appeared that the AFP level was in the course of the natural decline and abdominal ultrasonography showed no evidence of hepatic tumor mass. On the third visit (+17 weeks), the AFP level had slightly increased, but abdominal ultrasonography was not performed due to the parent's refusal. On the fourth visit (+29 weeks), his serum AFP level was elevated to >200,000 ng/ml and abdominal computer tomography scan revealed a well-defined heterogeneous liver mass (6.5 × 6.2 cm) (Figures 1A, 1B) suspicious of a hepatoblastoma. The hepatoblastoma was classified as PRETEXT stage II, a staging system for primary malignant liver tumors of childhood, that involved two sections (left medial and right anterior sections) of the four without any evidence of distant metastasis. He received transarterial chemoembolization with adriamycin and carboplatin, and neoadjuvant chemotherapy with cisplatin, vincristine and 5-fluorouracil. After 3 cycles of chemotherapy, hepatic segmentectomy (segment I, IV) was performed for curative surgery. The histopathologic diagnosis was hepatoblastoma with epithelial type (Figure 1C, 1D). He is now at complete remission state at 3 years post-operatively with five subsequent cycles of chemotherapy performed.

### Molecular-genetic basis of BWS diagnosis

**Figure 2:**
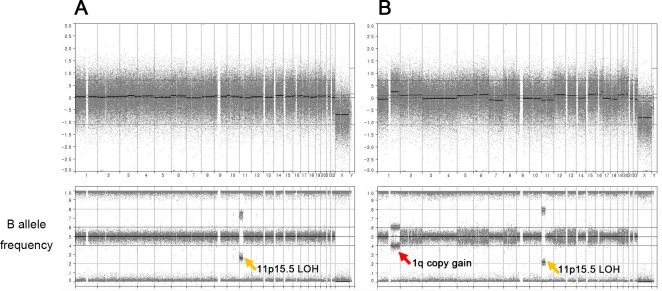
SNP array analysis of both normal **(A)** and tumor **(B)** genomes. Both samples show copy-neutral loss of heterozygosity at chromosome 11p15 (chr11:192,764-45,663,568) which suggests germline uniparental disomy as the genetic subtype of BWS (orange arrow). There is a somatic copy gain at chromosome 1q of the tumor genome (red arrow).

OncoScan SNP array analysis was performed to help the genetic diagnosis and molecular characterization of the BWS in this patient. Whole genome view with B-allele frequency for both normal (Figure 2A) and hepatoblastoma tumor tissue (Figure 2B) revealed copy-neutral LOH at chromosome 11p15.5 (chr11:192,764-45,663,568), indicating a germline UPD as a molecular subtype of BWS. When we estimated the UPD levels based on their B-allele frequencies, tumor tissue showed slightly higher UPD fraction (60%) than normal tissue (50%). There was a somatic copy number alteration (CNA) in the tumor genome (1q gain), while other chromosomes were largely stable (Figure 2B). The 1q gain has previously reported as the most frequent CNA in hepatoblastomas [11].

### Catalogue of somatic mutations

**Figure 3:**
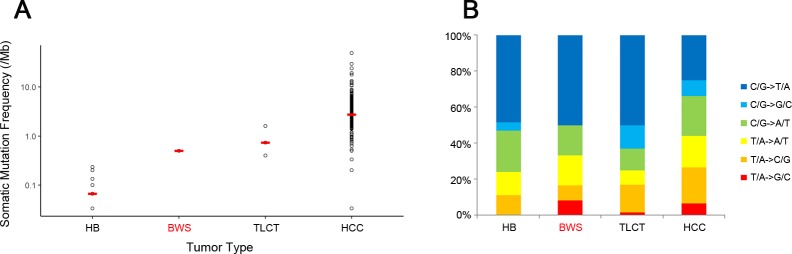
Comparison of somatic mutation frequency **(A)** and mutation spectra **(B)** occurring in BWS and other liver related cancers. Mean values of somatic mutation frequencies are represented with red bars in plot A. Abbreviations are: HB, hepatoblastoma; BWS, Beckwith-Wiedemann syndrome; TLCT, transient liver cell tumor; HCC, hepatocellular carcinoma

Mean coverage of the WES depth was 101X for tumor genome and 54X for normal, with an average of 93% of bases covered by at least 10 reads. After calling somatic mutations using MuTect and SomaticIndelDetector with default parameters, reads less than 20 were filtered out. After the filtering process, a total of 15 somatic mutations (12 point mutations and three indels) were called (Supplementary Table 1), corresponding to 0.5 somatic mutations per megabase (Mb), which was similar to those of previously reported for hepatoblastoma (0.067 per Mb) [10, 11] and TLCT (0.73 per Mb) [10], but much lower than that of adult HCC (TCGA provisional data, 2.73 per Mb) (Figure 3A). C:G to T:A transitions were predominant, consistent with the mutation spectra of liver cancers in adults and children [10, 13] (Figure 3B).

**Figure 4:**
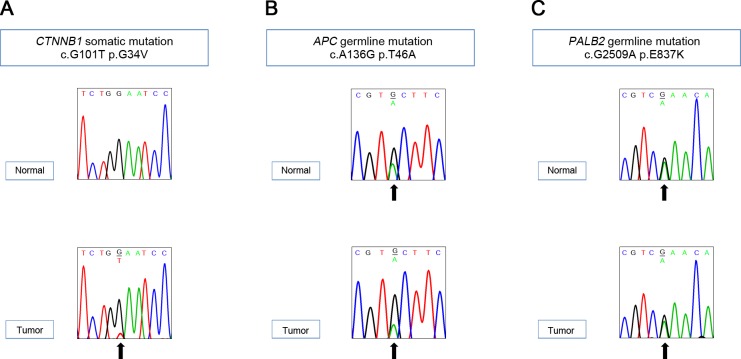
Sanger validation of the variants detected by whole exome sequencing *CTNNB1* somatic **(A)**, *APC* germline **(B)**, and *PALB2* germline **(C)** mutations.

Twelve non-silent gene mutations were detected: *CTNNB1*, *OVGP1*, *SCN2A*, *HCLS1*, *MUC4*, *MUC6 OR10S1*, *TNRC6A*, *SLC9A5*, *SUGP2*, *ZNF99*, and *ZNF606* (Supplementary Table 1). Of them, only *CTNNB1* was catalogued in the COSMIC cancer Gene Census, suggesting that the *CTNNB1* mutation might be a driver. *CTNNB1* c.G101T (p.G34V) somatic mutation was further confirmed with Sanger sequencing (Figure 4A). The *CTNNB1* p.G34V has been reported as a recurrent driver mutation occurring in hepatoblastoma [14] and in other human cancers [15]. Immunohistochemistry revealed β-catenin nuclear staining in hepatoblastoma cells, suggesting activated Wnt signaling in the tumor (Figure 1E). Functional annotation of the mutated genes using the DAVID database [16] revealed that the Wnt signaling pathway was the most associated biological process (Supplementary Table 2), indicating that the activation of the Wnt pathway may be the key driver in sporadic hepatoblastoma [9–11] and in BWS-related hepatoblastoma.

### Mutation signatures

**Figure 5:**
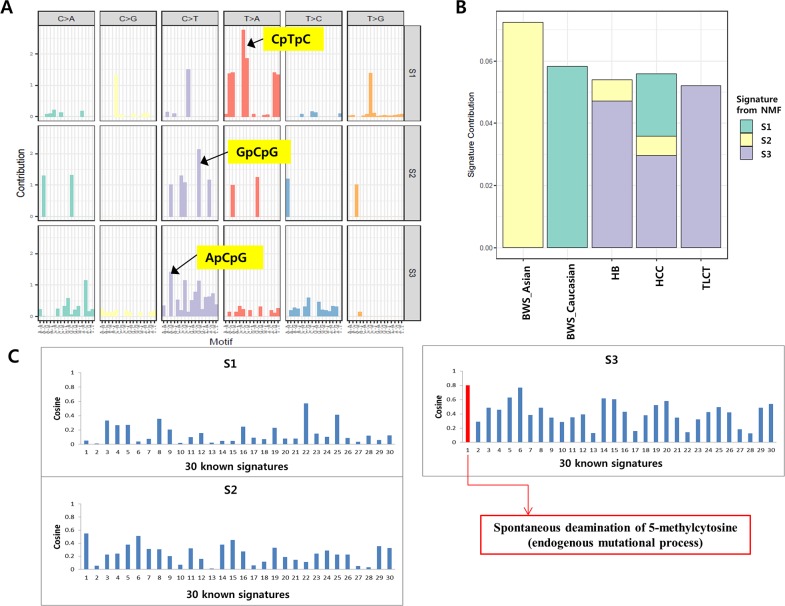
Mutation signatures of hepatoblastomas **(A)** Mutation spectra based on 96 base substitutions for the three signatures generated from NMF algorithm. **(B)** Proportion of the total substitutions contributed by each of the three mutational signatures (S1, BWS (Caucasian); S2, BWS (this case); S3, other liver tumors). **(C)** The graph shows similarities between three mutational signatures and 30 known mutation signatures from the COSMIC database. Red bars indicate high similarities with cosine similarity ≥ 0.8.

Mutational signature analyses showed that the hepatoblastoma with BWS in our study was dominated by the C>T transition in the context of GpCpG trinucleotides (Figure 5A and 5B). A previously reported hepatoblastoma with BWS (Caucasian) [9] was dominated by T>A transition in the context of CpTpC trinucleotides. The mutational signatures of other liver tumors (hepatoblastoma, HCC and TLCT) were dominated by C>T transition in the context of ApCpG trinucleotides (Figure 5A and 5B). Similarities between mutational signatures were calculated using a cosine correlation similarity which ranges between 0 and 1. Mutation signatures in sporadic hepatoblastoma exhibited the signature 1 of the 30 known signatures in the COSMIC database (cosine similarity ≥ 0.8), whereas those in the two hepatoblastomas with BWS (our case and the Caucasian BWS [9]) did not show any known signature (cosine similarity < 0.8) (Figure 5C).

### Germline mutations

We analyzed the WES data of the normal tissue genome to find any germline alterations possibly contributing to the predisposition to hepatoblastoma in BWS. Germline variants were called using the GATK HaplotypeCaller with default parameters. Synonymous variants and variants of more than 1% minor allele frequency in 1,000 Genomes Project or The Exome Aggregation Consortium (ExAC) database were excluded. Among the filtered variants listed, we searched for the genes that were catalogued in the COSMIC cancer Gene Census as known germline mutations predisposing to cancers (Supplementary Table 3) and identified a heterozygous *APC* missense mutation c.A136G (p.T46A), which was further validated with Sanger sequencing (Figure 4B). This mutation was identified in both normal and tumor tissues of the patient and was confirmed as a germline mutation. The parents of the BWS infant had neither family history of FAP nor clinical symptom of FAP. The *APC* p.T46A mutation was a novel one that had not been reported. In addition, we detected a germline mutation in *PALB2* gene c.G2509A (p.E837K) encoding a protein for double strand break repair (Figure 4C) [17]. The damaging scores of the mutants *APC* and *PALB2* assessed by both SIFT and MutationTaster were “tolerable” (Supplementary Table 3). There was no *CDKN1C* germline mutation known to be mutated in about 5-10% of sporadic BWS and in about 50% of familial BWS [18].

## DISCUSSION

The aim of this study was three-fold. First, we aimed to disclose somatic mutations in entire coding genes of a hepatoblastoma in a BWS patient. Second, we attempted to analyze genome-wide CNA and LOH profiles in that patient. Third, we attempted to find genomic alterations predisposing to BWS-related hepatoblastoma development besides chromosome 11p15.5 alterations. In a hepatoblastoma arising from this BWS infant, we report hitherto unknown germline and somatic genomic alterations. At somatic levels, previously known *CTNNB1* mutation and chromosome 1q gain were identified driver events, but no additional driver events were uncovered in the hepatoblastoma. In a previous WES of a hepatoblastoma with BWS, there was only a *CTNNB1* mutation in the tumor [9]. Together, it appears that there may not be additional somatic alterations in BWS-related hepatoblastoma compared to those in sporadic hepatoblastomas. At germline levels, we confirmed the genetic cause of BWS in the patient by identifying 11p15.5 UPD. In addition, we detected *APC* and *PALB2* missense germline mutations. PALB2 interacts and cooperates with BRCA2, and mediates DNA damage repair. *PALB2* is a tumor suppressor gene; its alteration of which was known to predispose to childhood Wilms’ tumor and medulloblastoma as well as breast cancers [19]. Of note, the *PALB2* germline mutation (p.E837K) identified in our study was previously reported in familial breast cancer [20]. Many reports have found the presence of *APC* germline mutations associated with hepatoblastoma especially in the kindreds of FAP [21, 22]. Even in patients with apparently sporadic hepatoblastomas, germline *APC* mutations are identified in about 10% [8]. Although the *APC* and *PALB2* germline mutations were not predicted as being pathogenic, further studies are necessary to conclude whether these two mutations play a synergistic role for the tumorigenesis of hepatoblastoma.

Analyses of the mutation signatures of our and a previous case [9] of hepatoblastomas with BWS showed that their signatures were distinct from sporadic hepatoblastomas. It is possible that the difference could come from the difference between genetic backgrounds (germline (BWS) and sporadic). However, the sample size was too small to conclude the hypothesis and further studies with a larger cohort of hepatoblastoma with BWS should be performed to clarify our observation. Children with BWS are at increased risk of tumors, therefore tumor surveillance particularly for Wilms’ tumor and hepatoblastoma is recommended for them [12]. We tried to follow the recommended tumor surveillance protocol of AFP and abdominal ultrasonography every 2-3 months for this patient. AFP level was checked according to the schedule, while ultrasonography was not, because the parents did not follow the instructions properly, which resulted in the delay of diagnosis of hepatoblastoma by two months. Our data strongly suggest that it is very important to follow the surveillance protocol strictly and to inform the parents of the possible consequences of the delay. Current data on the best schedule for the tumor surveillance are limited (2-3 months schedule interval for most recommendations) [23]. Several studies suggest the use of more intense protocols that adopt shortened intervals in early infancy, especially in babies with UPD or severe phenotypes [24, 25]. Even though the patient did not show severe phenotype of BWS, based on the genetic analysis, we can conclude that this baby was a high-risk patient and that strict and intense surveillance protocol (even shorter than 3 months) would have made the earlier diagnosis possible.

In summary, we found chromosome 11p15.5 UPD as a cause of BWS as well as germline mutations of *APC* and *PALB2* genes in a hepatoblastoma patient. Germline concurrence of 11p15.5 UDP, *APC* and *PALB2* in a hepatoblastoma is reported for the first time. As for somatic alterations, we detected a *CTNNB1* hotspot mutation and chromosome 1q gain, both of which were well-known somatic alterations in hepatoblastoma, suggesting a pivotal role for development of the hepatoblastoma. To our knowledge, this is the first report of germline and somatic genomic alteration profiles in hepatoblastoma arising from BWS. The development of hepatoblastoma in this BWS infant can be explained by predisposition of UPD at 11p15.5 as well as possibly of *APC* and *PALB2* mutations and later by somatic events with *CTNNB1* somatic mutation and 1q gain acting as driver alterations. Clinically, our study provides a rationale for performing a more strict and intense protocol for hepatoblastoma surveillance in the UPD-carrying BWS infant.

## MATERIALS AND METHODS

### Tissue sampling

Formalin-fixed paraffin-embedded (FFPE) tumor tissue of the hepatoblastoma was obtained from Seoul St. Mary's Hospital of the Catholic University (Seoul, Korea). Informed consent from the parents and approval for this study were obtained from the Institutional Review Board of the Catholic University of Korea, College of Medicine. FFPE tumor tissue and adjacent normal liver tissue (hepatitis (-) and cirrhosis (-)) were manually microdissected by a pathologist. Genomic DNA was prepared using GeneRead DNA FFPE Kit (Qiagen, Hilden, Germany).

### Whole-exome sequencing and variant calling

WES analyses for both tumor and normal genomic DNA were performed using the Agilent SureSelect Human All Exome 50Mb Kit (Agilent Technologies, Santa Clara, CA) and Illumina HiSeq2000 platform according to the manufacturer's instructions. 101 bp paired-end reads were generated using the Illumina HiSeq2000 platform and the Burrows-Wheeler aligner was used to align the sequencing reads onto the human reference genome (hg19). Somatic variants were identified using MuTect [26] and SomaticIndelDetector [27] for point mutations and indels, respectively. Germline variants were identified using GATK HaplotypeCaller. All variants were annotated using ANNOVAR [28]. Mutation rates and spectra were compared with previously reported studies of hepatoblastoma [10, 11], transient liver cell tumor (TLCT) [10], and adult hepatocellular carcinoma (HCC) from The Cancer Genome Atlas (TCGA).

### DNA copy number and loss of heterozygosity analyses

Single nucleotide polymorphism (SNP) array analysis for both normal and tumor genomic DNA was performed using OncoScan FFPE Assay Kit (Affymetrix, Santa Clara, CA). The DNA copy number and loss of heterozygosity (LOH) were analyzed and visualized using the SNP-FASST2 Segmentation statistical algorithm in NEXUS software v7.5 (Biodiscovery Inc., El Segundo, CA). All of the identified CNAs and LOH events were manually curated in terms of log_2_ ratio and B allele frequency.

### Mutational signature analysis

Mutational signature analyses were conducted using SomaticSignatures [29] R package which estimates somatic signatures with a non-negative matrix factorization algorithm. We analyzed mutational signatures of hepatoblastomas including our BWS patient, previously reported hepatoblastoma [10, 11], TLCT [10], adult HCC from The Cancer Genome Atlas (TCGA), and a recently reported Caucasian BWS patient [9]. Similarities between mutational signatures were calculated using a cosine correlation similarity method with 30 known signatures recorded in the Catalogue of Somatic Mutations in Cancer (COSMIC) database [30].

## SUPPLEMENTARY MATERIALS FIGURES AND TABLES








