# Supplementary material for: Genomic profiles of a hepatoblastoma from a patient with Beckwith-Wiedemann syndrome with uniparental disomy on chromosome 11p15 and germline mutation of APC and PALB2
Source: Oncotarget. 2017 Aug 24;8(54):91950–7. doi: 10.18632/oncotarget.20515 (PMC5696154; doi:10.18632/oncotarget.20515)
Supplement: Supplementary file 1 [file oncotarget-08-91950-s001.pdf]

Genomic profiles of a hepatoblastoma from a patient with Beckwith-Wiedemann syndrome with uniparental disomy on chromosome 11p15 and germline mutation of APC and PALB2

SUPPLEMENTARY MATERIALS

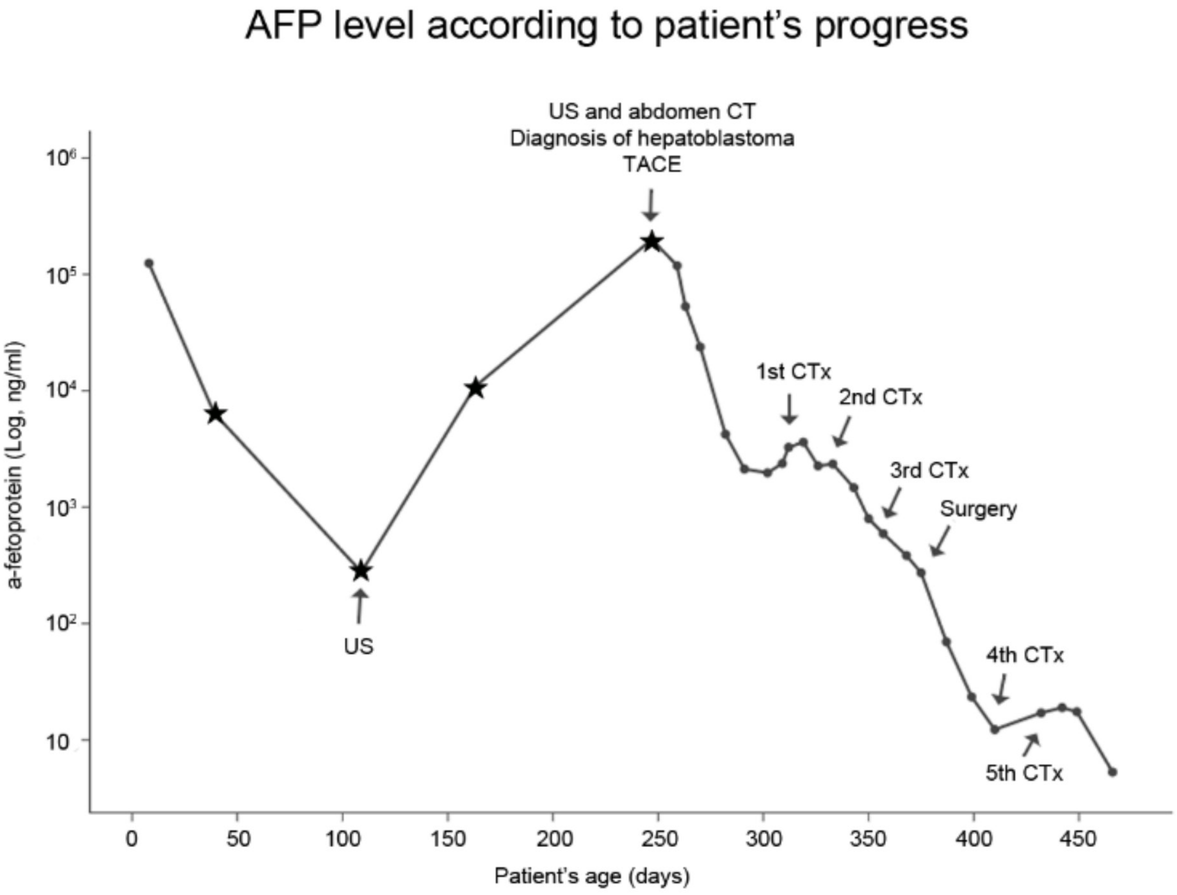

Supplementary Figure 1: Serum α-fetoprotein level from birth to the end of treatment with major events during the course.

Supplementary Table 1: Somatic mutations identified by whole exome sequencing of Beckwith-Wiedemann syndrome patient.

See Supplementary File 1

Supplementary Table 2: Gene ontology analysis of somatic mutations using DAVID (http://david.abcc.ncifcrf.gov/) listed according to significance.

See Supplementary File 2

Supplementary Table 3: Germline variants of the patient catalogued in the COSMIC cancer Gene Census.

See Supplementary File 3
